# Double-Inverse-Opal-Structured Particle Assembly as a Novel Immobilized Photocatalytic Material

**DOI:** 10.3390/ma14010028

**Published:** 2020-12-23

**Authors:** Hikaru Namigata, Kanako Watanabe, Saya Okubo, Masashi Hasegawa, Keishi Suga, Daisuke Nagao

**Affiliations:** Department of Chemical Engineering, Tohoku University, Sendai 980–8579, Japan; hikaru.namigata.r1@dc.tohoku.ac.jp (H.N.); kanako.w@tohoku.ac.jp (K.W.); okubo0706@gmail.com (S.O.); masashi.hasegawa.s6@dc.tohoku.ac.jp (M.H.); keishi.suga.b7@tohoku.ac.jp (K.S.)

**Keywords:** photocatalyst, titanium dioxide, particle assembly, double-inverse-opal, immobilization, flow reaction

## Abstract

Immobilization of photocatalysts on supports is an important method of adding highly active photocatalysts to a continuous flowing system without the need for photocatalyst recovery. However, direct immobilization prevents exposure to all photocatalytically active surfaces. Therefore, to immobilize particulate photocatalysts, while exposing the photocatalytic surface to organic pollutant water in a continuous flowing system, in this study, we employed double-inverse-opal (DIO) with periodically arranged, interconnected macropores, each containing a single photocatalytic particle. Increasing the macropore size successfully enhanced the decomposition rate of organic dye due to the high diffusion rate of dye molecules in the macropores of thin DIOs. However, an excessive increase in macropore size lowered the decomposition rate of dye molecules because an increase in DIO thickness caused the attenuation of light used to excite the photocatalytic particles. This study presents novel, immobilized photocatalytic DIO-structured particles that can be employed in continuous flowing reaction systems by tuning the photocatalytic particle size, macropore size, and DIO thickness.

## 1. Introduction

Photocatalysts that promote oxidation-reduction reactions using light energy have attracted significant attention, owing to their applications in various fields, such as the decomposition of organic pollutants [1,2,3], production of hydrogen [4,5,6], and development of solar cells [7,8]. Crystalline TiO_2_ has been widely used as a semiconductor photocatalyst since the “Honda–Fujishima effect [4]” was reported in the 1970s. The bandgap of crystalline TiO_2_ depends on its crystal structure (3.2 eV for anatase and 3.0 eV for rutile [9]). These bandgaps correspond to ultraviolet (UV) light. To effectively excite TiO_2_ photocatalysts using visible light, TiO_2_ must be functionalized or doped with visible light-responsive materials such as metal nanoparticles [10,11,12], nitrogen, and sulfur [13,14]. Alternative photocatalysts such as CdS [15], BiVO_4_ [16,17], and g-C_3_N_4_ [18,19] have also been studied in recent years. TiO_2_-utilized photocatalyst systems, however, have been extensively researched, owing to their stability and safety.

Downsizing of photocatalysts to the submicron range can be employed for particulate photocatalysts to avoid electron–hole recombination while also increasing their surface area [20]. In batch processes, particulate photocatalysts that are colloidally stable offer a large surface area that can be exposed to reactants under irradiation. However, to recycle these photocatalysts, the dispersion system requires separation and recovery processes [1,21], increasing the cost of the photocatalytic process in industrial applications. Therefore, photocatalysts immobilized on inert supports [22,23] (“immobilized-type” photocatalysts) that do not require a separation process have been studied as an alternative. These include inverse-opal (IO) arrays [24] and photocatalytic fiber mats [25], which are suitable for use in continuous processes in a flow reactor. However, direct immobilization onto supports causes the surface of the photocatalysts to be coated with the support material, leading to a decrease in photocatalytically active surface area [26]. Therefore, it is necessary to develop a system that confines particulate photocatalysts to a specific region without decreasing their active surface area.

In this study, a double-inverse-opal (DIO) photocatalytic structure is proposed as a novel, immobilized-type photocatalyst system. The DIO structure [27,28,29] consists of periodically ordered macropores containing a single particle within the macropore framework. The DIO is a hierarchically structured material developed as an advanced inverse opal that is applicable to separation materials and photonic crystals [30,31]. This novel DIO has ordered macropores containing a TiO_2_ sphere that comes into contact with reactant solution in the macropore framework. TiO_2_ is a highly processable material, suitable for preparing spherical photocatalysts. Silica was chosen as the DIO frame because it has high mechanical strength [32] and exhibits low UVA (320–400 nm) and visible light absorption [33,34]. The DIO photocatalytic assemblies were fabricated via a simple method of self-assembly of template particles. The photocatalytic activity of the DIO assembly was examined using a model photocatalytic reaction in a quasi-continuous system. Herein, the effects of certain DIO assembly parameters, such as the size of macropores and the thickness of assembly, on the catalytic activity are investigated.

## 2. Materials and Methods

### 2.1. Materials

Titanium tetraisopropoxide (TTIP, 95%), methylamine aqueous solution (40%), acetonitrile (99.5%), ethanol (99.5%), styrene (St, 99%), *p*-styrenesulfonic acid sodium salt (NaSS, 80%), potassium persulfate (KPS, 95%), sodium chloride (99.5%), tetraethyl orthosilicate (TEOS, 95%), hydrochloric acid (HCl, 0.10 mol/L), and methylene blue (MB, 98.5%) were purchased from FUJIFILM Wako Pure Chemical Corp. (Osaka, Japan). 3-Methacryloxypropyltrimethoxysilane (MPTMS, 95%) was obtained from Shin-Etsu Chemical Co. (Tokyo, Japan). The inhibitor for the St monomer was removed using an inhibitor removal column. Deionized water (>18.2 MΩ cm) was prepared for experimental use.

### 2.2. Fabrication of the DIO Photocatalytic Assembly

**Figure 1:**
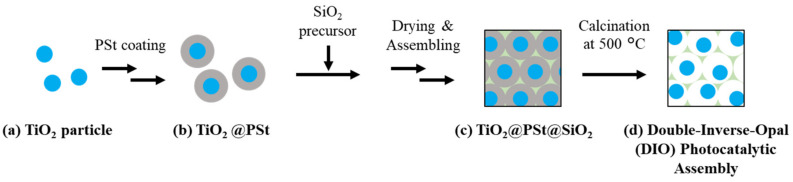
Schematic procedure of double-inverse-opal (DIO) structure with a combined component of TiO_2_ particles and inverse opal SiO_2_ frame. (**a**) Synthesis of TiO_2_ core particles, (**b**) Polystyrene-coating of TiO_2_ particles (TiO_2_@PSt), (**c**) Filling the interstices of assembled TiO_2_@PSt with SiO_2_ (TiO_2_@PSt@SiO_2_), and (**d**) Removal of polystyrene shell (double-inverse-opal photocatalytic assembly).

Figure 1 describes preparation of the DIO photocatalytic assembly. Submicron-sized amorphous TiO_2_ cores were prepared via a sol–gel method, with a solvent containing ethanol and acetonitrile [35]. The TiO_2_ cores were coated with polystyrene (PSt), similar to our previous reports (Figure 1b) [36,37]. The shell thickness of the PSt formed on the cores ranged from 140 to 330 nm. The detailed synthesis protocol of the TiO_2_@PSt particles is presented in the Supplementary Material. After synthesis, the TiO_2_@PSt particles were assembled and the interstices between the PSt shells were filled with SiO_2_ precursors to be solidified to a SiO_2_ frame upon drying (Figure 1c). In the final step, the TiO_2_@PSt assembly with SiO_2_ frame was calcined to remove the PSt shells and crystallize the TiO_2_ cores (Figure 1d). To prepare the SiO_2_ frame precursor, we stirred TEOS and HCl together for 5 min at room temperature, and an aqueous suspension of TiO_2_@PSt and ethanol was added to the mixture. The volume fraction of TiO_2_@PSt in the mixture was 0.25 vol %. The concentrations of TEOS and HCl were 25 and 0.5 mM, respectively. Subsequently, 200 µL of the mixture was poured onto a glass substrate (*ϕ* = 18 mm, Matsunami Glass Ind. (Osaka, Japan), C018001), which had been hydrophilized with a UV ozone cleaner (Filgen, Aichi, Japan, UV253E) for 30 min. After the dispersion medium of the mixture was evaporated, the particle assembly was calcined for 2 h at 500 °C. Notably, the TiO_2_ particles of the assemblies could be crystallized into the anatase phase via heat treatment at 500 °C [37,38].

### 2.3. Characterization of the DIO on Photocatalytic Activities

The photocatalytic activity of the DIO assemblies was examined in an experiment where an organic dye MB [39,40] was decomposed in a circulating system of aqueous solution under UV irradiation. The assemblies were placed in a cell with dimensions of *ϕ* = 28 mm and height = 2 mm (S.T. Tokyo, Japan), 162–1100). As shown in Figure S1, the container with the MB aqueous solution was connected to one side of the cell by a plastic tube. On the other side of the cell, the tube pump (AS ONE, FP-300–1515) was connected to circulate the MB solution at a flow rate of 20 mL/min. To obtain uniform adsorption of MB on the surface of the TiO_2_ particles, we conducted the circulation of the MB solution for 1.5 h in the dark (without UV light irradiation). It was confirmed that the adsorption equilibrium of MB was attained within 1.5 h in this preliminary experiment (see Figure S2). Thereafter, the initial concentration of MB (*C*_0_) was examined on the basis of the absorption peak intensity at 664 nm, which was measured using a UV–VIS spectrophotometer (Hitachi, Tokyo, Japan, U-3900). The photocatalytic reaction was initiated by the irradiation of the assembly with UV light (*λ* = 365 nm, 0.50 mW/cm^2^). After initiating UV irradiation, we withdrew 1.5 mL of the solution from the reactor at various times throughout the reaction (0.5–5 h), and we determined the residual MB concentration (*C*) using the 664 nm peak. A xenon lamp (Asahi Spectra, Tokyo, Japan, MAX-303) with a band-pass filter (Asahi Spectra (Tokyo, Japan), HQBP-UV *ϕ*25) that shields visible light was used as the UV light source. During the reaction, the MB solution was continuously circulated at a flow rate of 20 mL/min, a figure chosen due to the results of a preliminary photocatalytic experiment (see Figure S3). It was found that a higher flow rate, 30 mL/min, peeled the DIO assembly from the glass substrate.

### 2.4. Characterization

The obtained particles and particle assemblies were observed via field-emission scanning transmission electron microscopy (FE-TEM, Hitachi, Tokyo, Japan, HD-2700) and field-emission scanning electron microscopy (FE-SEM, Hitachi, Tokyo, Japan, S-4800), respectively. The transmittance of the particle assemblies was measured using the UV–VIS spectrophotometer mentioned previously. The volume-averaged diameter (*d*_V_) was determined using the following equation:

(1) $$d_{V}={\left(\sum n_{i}d_{i}{}^{3}/\sum n_{i}\right)}^{1/3}$$

where $n_{i}$ is the number of particles with a diameter $d_{i}$, and *d*_V_ was determined by measuring the diameters of more than 200 particles using TEM images. The size of the macropores in the DIO assemblies *(d*_p_) was estimated by direct measurement of macropores in SEM images. The average thickness of the assemblies (*T*) was determined by measuring more than 50 spots in the cross-sectional SEM images of each assembly.

## 3. Results and Discussion

### 3.1. Photocatalytic Activity of the DIO Photocatalytic Assembly

**Figure 2:**
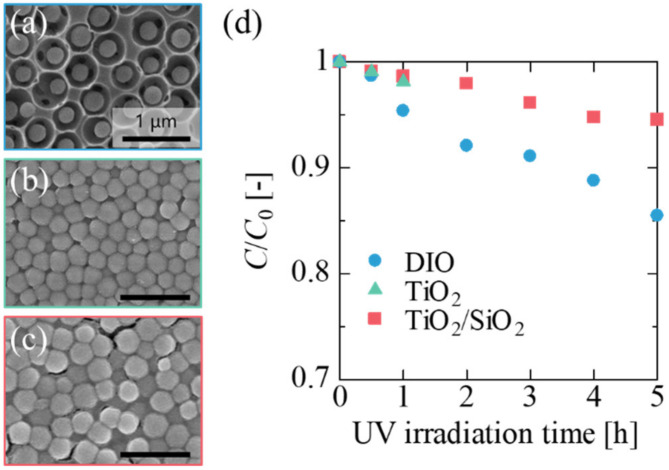
SEM images of (**a**) DIO photocatalytic assembly, (**b**) TiO_2_ particle assembly, and (**c**) TiO_2_ particle assembly immobilized by SiO_2_ frame (TiO_2_/SiO_2_). (**d**) Photocatalytic activities of DIO assembly, TiO_2_ particle assembly, and TiO_2_/SiO_2_.

An SEM image of the DIO photocatalytic assembly is shown in Figure 2a. The DIO assembly comprises macropores with an ordered structure, with each macropore containing a single TiO_2_ particle. *d*_V_ and *d*_p_ were 400 and 240 nm, respectively, indicating that the TiO_2_ particles and the frame surface were separated by an average distance of 80 nm. As shown by a magnified SEM image (Figure S4), the voids are connected via holes with a diameter of approximately 100 nm, which are called interconnecting pores [30,41]. The holes play an important role in forming channels for the reactant solution in the DIO assembly. The size of the interconnecting pores was much smaller than that of the TiO_2_ particles, indicating that the particles were unable to move to adjacent macropores or to outside the assembly. The transmission spectrum of the DIO assembly in the visible light and UVA region (350–700 nm) is shown in Figure S5. In the visible light region, the transmittance was lower than those of glass substrate and a SiO_2_ inverse opal frame because of scattering by the TiO_2_ particles within the DIO. In the UV region, the transmittance of the DIO assembly was lower than it was in the visible light region. This suggests that the TiO_2_ particles inside the DIO assembly can be excited through UV light absorption.

The photocatalytic activity of the DIO assembly was examined on the basis of the decomposition of MB under UV irradiation. To investigate the effect of voids (i.e., the space between the TiO_2_ core and the SiO_2_ frame) on the photocatalytic activity of the DIO assembly, we fabricated a TiO_2_ particle assembly (TiO_2_ in Figure 2b) and a TiO_2_ particle assembly immobilized by a SiO_2_ frame without voids (TiO_2_/SiO_2_ in Figure 2c) were fabricated and their photocatalytic activities were compared with those of the DIO assembly. Figure 2d shows the MB concentrations *(C*/*C*_0_) plotted against irradiation time for each assembly. The TiO_2_ particle assembly was highly fragile; under the flow of MB aqueous solution, the assembly collapsed within 2 h of initiating UV irradiation, and almost all the assembled particles flowed out of the substrate. In contrast, the TiO_2_/SiO_2_ assembly was sufficiently strong, withstanding the flow of the MB solution for 5 h, owing to immobilization with the SiO_2_ frame. The MB molecules in the solution were initially decomposed, similar to those in the solution employed for the TiO_2_ assembly, indicating almost no contact between the MB molecules and the TiO_2_ interface in the assemblies. The DIO assembly developed for the continuous photocatalytic system possessed a mechanical strength similar to that of the TiO_2_/SiO_2_ assembly and exhibited a higher activity than those of both the TiO_2_ and TiO_2_/SiO_2_ assemblies.

The superiority of the novel DIO assembly could be attributed to the effective surface area of the TiO_2_ particles compartmentalized in macropores. As for the TiO_2_/SiO_2_ assembly, the interface of the TiO_2_ particles inside the assembly cannot be used for photocatalytic reactions because the particles are closely packed, with the particle interstices filled with SiO_2_. In contrast, the surface of the TiO_2_ particles in the DIO assembly were exposed to the MB solution flowing from each macropore to its neighboring ones through the interconnecting pores. In fact, the DIO assembly exhibited greater MB adsorption than that of the TiO_2_ assembly and the TiO_2_/SiO_2_ assembly, indicating a larger effective surface area (see Table S1).

### 3.2. Effect of Void Size and Film Thickness on Photocatalytic Activity

**Figure 3:**
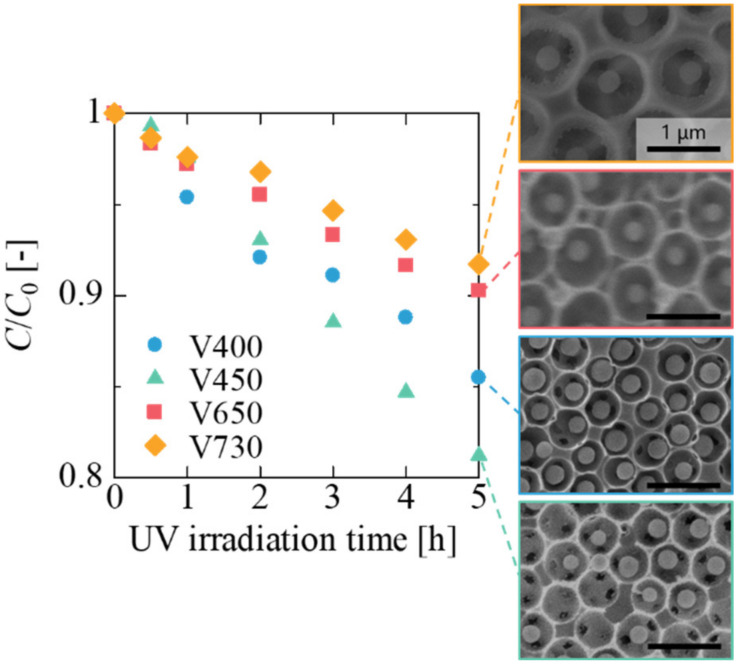
Photocatalytic activities of DIO assemblies with different diameters of macropores. V400 (*d*_p_ = 400 nm, blue dots), V450 (*d*_p_ = 450 nm, green triangles), V650 (*d*_p_ = 650 nm, red squares), V750 (*d*_p_ = 730 nm, yellow rhombuses). SEM images on the right are top views of each structure.

DIO photocatalytic assemblies with four different diameters (*d*_p_) of macropores were prepared by tuning the PSt shell thickness on a TiO_2_ core. The variation in the thickness of the PSt shell increased the macropore diameter from 400 to 730 nm. In Figure 3, where macropore size is indicated by the sample names (V400, V450, V650, and V730), the decrease in MB concentration during the photocatalytic reaction for the four DIOs are presented. Comparing the residual concentrations of MB for a reaction time of 5 h, the highest decomposition rate was observed in V450, followed by V400, V650, and V730 (Table S2 shows the rate constants for the photocatalytic reaction of each DIO assembly). To discuss the difference in photocatalytic activity, the space between the TiO_2_ core and the SiO_2_ frame, which simultaneously affects the diffusion of the MB molecules and the propagation of UV light in a macropore, should be considered.

The larger spaces in V450, when compared with V400, for the diffusion of MB could prolong the presence of MB in the former, thus offering a high frequency of contact between the MB and the TiO_2_ surface. However, the large spaces resulted in thicker DIO assemblies, owing to the same number of TiO_2_ particles (approximately 4.3 × 10^10^ unit for each) incorporated in them; the thickness of the DIO assemblies increased from 7.2 µm for V400 to 29 µm for V730. The increase in thickness of the DIO assemblies attenuates the irradiation because of the increased optical length. To examine the two factors separately, we fabricated DIO assemblies with varying thicknesses using core-shell particles with similar PSt shells.

**Figure 4:**
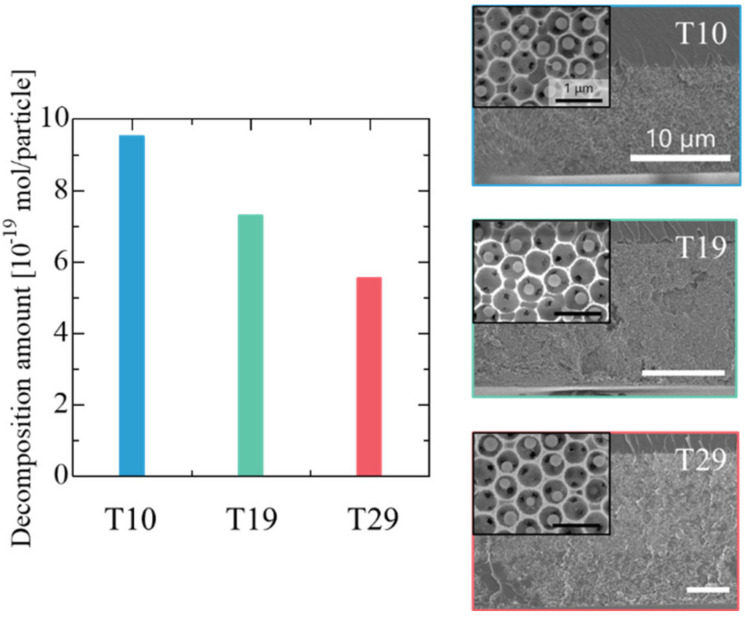
Decomposition amounts of methylene blue (MB) per a single TiO_2_ particle of T10 (*T* = 10 µm), T19 (*T* = 19 µm), and T29 (*T* = 29 µm) 5 h after the photocatalytic reaction started. SEM images on the right are cross sections and top views (inset) of each structure.

The SEM images on the right side of Figure S6 show DIO assemblies with thicknesses of 10, 19, and 29 µm (T10, T19, and T29, respectively), which were fabricated via iterative coating of a solution of core-shell particles and SiO_2_ precursor. A macropore diameter of 450 nm was confirmed by direct measurement of their sizes in SEM images. The photocatalytic activities of the DIO assemblies are shown on the left side of Figure S6; the thicker DIO assemblies decomposed larger amounts of MB. This was because the number of TiO_2_ components in the assembly increased with an increase in the thickness of the DIO. The amount of MB molecules that decomposed for each particle are presented in Figure 4 and the decomposition amount of MB was not proportional to the film thickness (see Figure S7 for the total amount of MB decomposed). The average amount of decomposed MB molecules for a single TiO_2_ particle in T10, T19, and T29 were 9.5 × 10^−19^, 7.3 × 10^−19^, and 5.6 × 10^−19^ mol/particle, respectively. This suggests the inhomogeneous excitation of the TiO_2_ particles due to the attenuation of light propagation in the thick DIO assemblies. A similar trend of non-proportionality in the decomposition rate for photocatalyst-supported films has been reported [42,43]. The transmittance intensity of light through the DIO assemblies is shown in Figure S8, which decreased with DIO thickness.

When the photocatalytic activity was normalized by TiO_2_ particle number, the decomposition amount of MB in V730 with a DIO thickness of 29 μm (Figure 3) was 5.3 × 10^−19^ mol/particle, which was very similar to that of T29 (5.6 × 10^−19^ mol/particle). This indicates that the thickness of the DIO assemblies is an important factor in determining the decomposition rate of the MB molecules, and the time spent inside the macropores is less important in DIOs with sufficient space for MB diffusion.

On the basis of the results, an increase in the macropore size in this novel DIO assembly could promote the diffusion of reactants; however, it simultaneously attenuated the incident light meant to excite the TiO_2_ cores in the DIO. A precise design—for the efficient use of light energy—is required for the development of a continuous photocatalytic system that is practical for industry use (e.g., precise control over the size of the TiO_2_ particles and macropores, the number density of the TiO_2_ particles, and film thickness). Another possible option is the employment of the “slow photon effect”. This is a phenomenon that slows down the propagation of light with wavelengths near the photonic band gap in certain photonic crystals [24,44]. Therefore, it is expected to enhance the photocatalytic activities in the DIO assemblies.

## 4. Conclusions

In this study, a DIO photocatalytic assembly of crystallized TiO_2_ particles encapsulated within macropores of a SiO_2_ frame was developed as a new type of immobilized photocatalyst. The fabricated DIO assembly exhibited higher photocatalytic activity than that of TiO_2_ particles immobilized within the SiO_2_ frame without voids. The SiO_2_ frame played an important role in preventing TiO_2_ particles from flowing out of the assembly. The interconnecting pores between the macropores provided a channel for the reactant solution to flow from one macropore to another with a single TiO_2_ particle. DIO assemblies with large macropores can increase the contact frequency of the reactant molecules with the photocatalytic surface exposed in the assembly. However, the increase in macropore size causes thickening of the DIO assembly, resulting in the inhomogeneous excitation of TiO_2_ particles in the assembly caused by attenuation of light propagation. This novel DIO offers a new type of immobilized photocatalytic structure to be applied to continuous photocatalytic reaction systems. To optimize the performance of DIO assembly system, the following factors should be considered: the photocatalytic particle sizes, macropore sizes, and film thickness.

## Supplementary Materials

The following are available online at https://www.mdpi.com/1996-1944/14/1/28/s1: Figure S1: Schematic illustration of the photocatalytic reactor. Figure S2: MB adsorption on TiO_2_ particles in the DIO photocatalytic assembly in dark condition. Figure S3: Photocatalytic activities of the DIO assembly with various flow rates. Figure S4: The SEM image of interconnecting pores between void spaces. Figure S5: Transmission spectra of a glass substrate, a SiO_2_ inverse opal, and the DIO photocatalytic assembly. Figure S6: Photocatalytic activities of DIO assemblies with different thicknesses. Figure S7: The total decomposition amount of MB versus film thicknesses. Figure S8: The transmittance versus thickness of DIO photocatalytic assemblies. Table S1: Adsorption amounts of MB on the TiO_2_ particle assembly, the TiO_2_ particle assembly immobilized by silica frame (TiO_2_/SiO_2_), and the DIO assembly. Table S2: MB decomposition rate constant of each DIO assembly.
