# Supplementary material for: Double-Inverse-Opal-Structured Particle Assembly as a Novel Immobilized Photocatalytic Material
Source: Materials (Basel). 2020 Dec 23;14(1):28. doi: 10.3390/ma14010028 (PMC7793507; doi:10.3390/ma14010028)
Supplement: Supplementary file 1 [file materials-14-00028-s001.zip › materials-1010251-supplementary.pdf]

Supplementary Material

Double-inverse-opal-structured particle assembly as a novel immobilized photocatalytic material

*Hikaru Namigata, Kanako Watanabe, Saya Okubo, Masashi Hasegawa, Keishi Suga and Daisuke Nagao**

* E-mail: dnagao@tohoku.ac.jp, TEL: +81 22-794-7240

Synthesis of TiO_2@PSt

The TiO_2 cores were coated with polystyrene (PSt) according to our previous reports (Figure 1b) [2,3]. TiO_2 particles and a silane coupling agent 3-mercaptopropyl trimethoxysilane (MPTMS) were added in a mixed solvent of ethanol and water. After stirring for 1 h at 35 °C, a monomer (St) and a comonomer (NaSS) were added, and the mixture was stirred further for 1 h. An aqueous solution of polymerization initiator potassium persulfate (KPS) was added to the mixture at 65 °C. TiO_2 particles coated with thin PSt shells were obtained 16 h after initiation of the polymerization. The volume fraction of TiO_2 particles in the mixture was 0.065 vol %. The concentrations of MPTMS, St, NaSS, and KPS were 2.4, 50, 2.5, and 2 mM, respectively.

In order to increase PSt shell thickness, we conducted further polymerization. St monomer and sodium chloride were added to the aqueous suspension of the TiO_2 particles with thin PSt shells (0.05 vol %). After stirring for 1 h at 35°C, we added an aqueous solution of KPS to the mixture and stirred the mixture for 16 h at 65°C. Obtained particles (TiO_2@PSt) were collected by centrifugation and redispersed in water. The concentrations of St and KPS were 100 mM and 2 mM, respectively. Sodium chloride at different concentrations (0, 1, 2, and 4 mM) was added to increase the thickness of PSt shells.

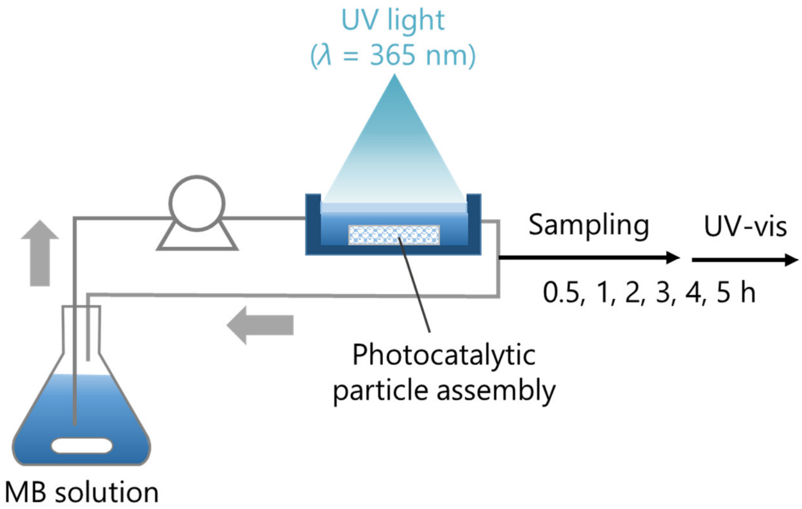

Figure S1. Schematic illustration of the photocatalytic reactor.

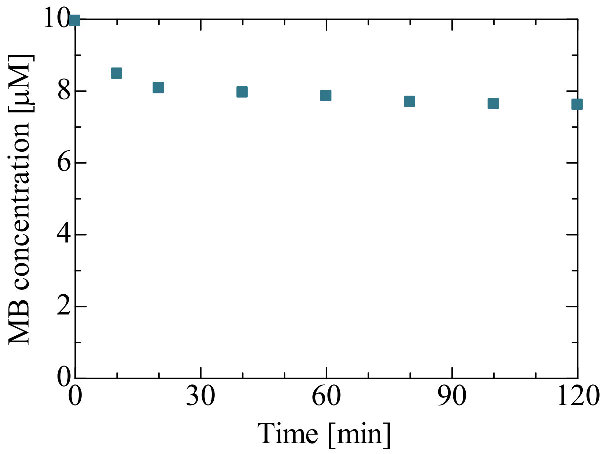

Figure S2. MB adsorption on TiO_2 particles in the DIO photocatalytic assembly in dark condition (flow rate = 20 mL/min).

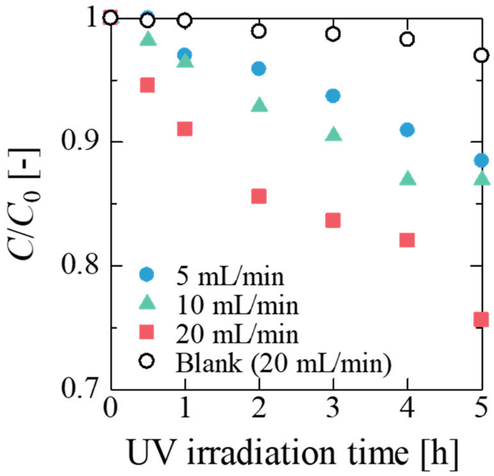

Figure S3. Photocatalytic activities of DIO with different flow rates: 5 mL/min (blue dots), 10 mL/min (green triangles), 20 mL/min (red squares), 20 mL/min without DIO (white dots).

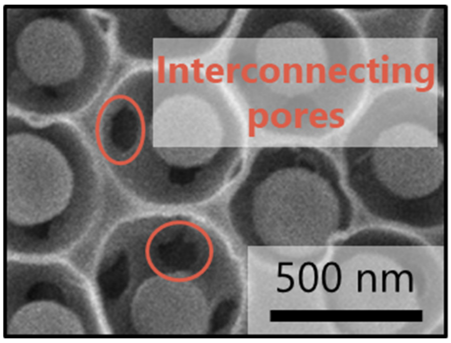

Figure S4. The magnified image of the SEM image shown in Figure 2a: interconnecting pores.

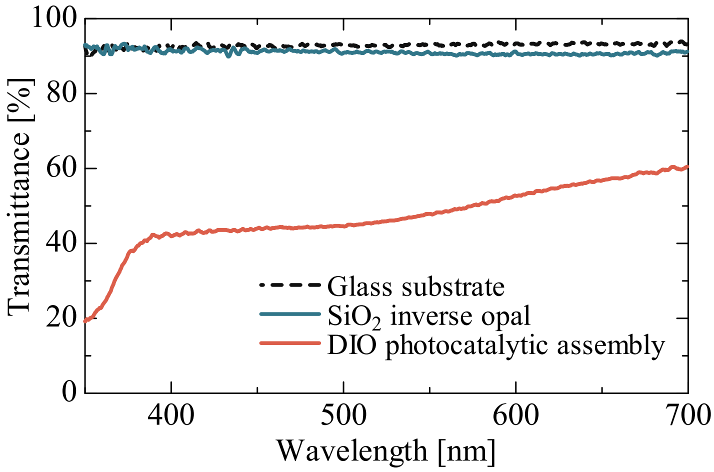

Figure S5. Transmission spectra of a glass substrate, a SiO_2 inverse opal, and the DIO photocatalytic assembly (V400).

Table S1. Adsorption amounts of MB on the TiO_2 particle assembly, the TiO_2 particle assembly-immobilized by silica frame (TiO_2/SiO_2), and the DIO assembly.

	ADSORPTION AMOUNT OF MB (MOL)
**TIO_2**	3.9×10^{-8}
**TIO_2/SIO_2**	4.5×10^{-8}
**DIO ASSEMBLY**	1.2×10^{-7}

Calculation of reaction rate constants

MB concentrations normalized by the initial concentration (C/C_0) were calculated using the Langmuir–Hinshelwood equation as follows.

$$-\ln(C/C_0) = kt$$

where k (h^{-1}) and t (h) are the reaction rate constant and time of the light irradiation, respectively.

Table S2. MB decomposition rate constant of each DIO assembly.

DIO Assemblies	k (h^{-1})
V400	3.2×10^{-2}
V450	4.1×10^{-2}
V650	2.2×10^{-2}
V730	1.8×10^{-2}
T10	3.2×10^{-2}
T19	6.9×10^{-2}
T29	8.0×10^{-2}

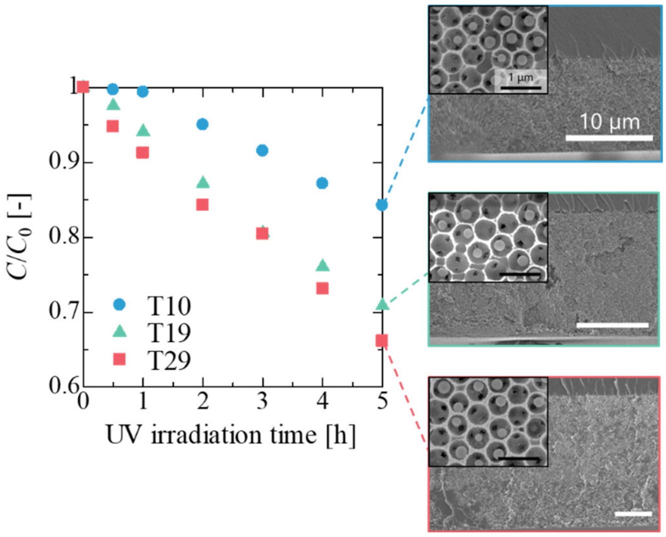

Figure S6. Photocatalytic activities of DIO assemblies with different thickness. T10 (H = 10 μm, blue dots), T19 (H = 19 μm, green triangles), and T29 (H = 29 μm, red squares). SEM images on the right are cross sections and top views (inset) of each structure.

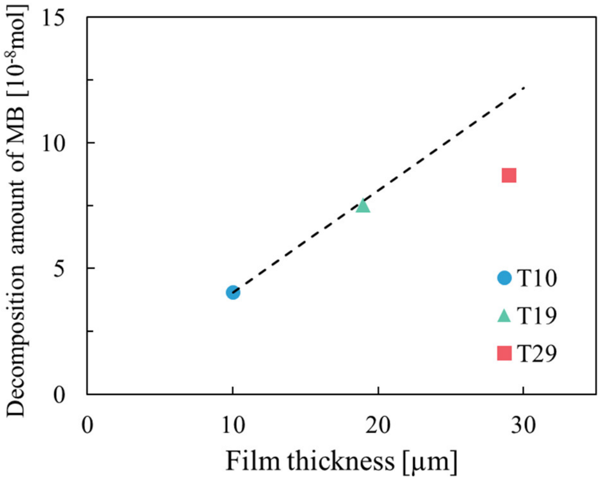

Figure S7. The total decomposition amount of MB by T10, T19, and T29 versus film thicknesses.

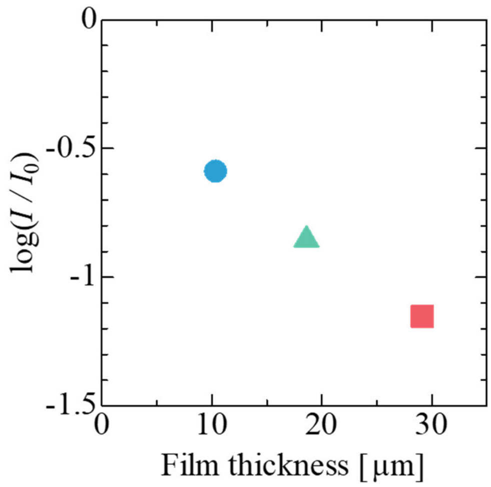

Figure S8. The logarithm of transmittance versus the thickness of DIO photocatalytic assembly (T10, T19, and T29).
